# Social accountability as a strategy to promote sexual and reproductive health entitlements for stigmatized issues and populations

**DOI:** 10.1186/s12939-021-01597-x

**Published:** 2022-02-10

**Authors:** Marta Schaaf, Grady Arnott, Kudzai Meda Chilufya, Renu Khanna, Ram Chandra Khanal, Tanvi Monga, Charles Otema, Christina Wegs

**Affiliations:** 1 Independent Consultant, 357 Sixth Ave., Brooklyn, NY 11215 USA; 2 Center for Reproductive Rights, Global Advocacy Program, 199 Water Street, 22nd Floor, New York, NY 10038 USA; 3 Ipas/Zambia, P.O Box 320402, Lusaka, Zambia; 4 SAHAJ (Society for Health Alternatives), 1 Shri Hari Apartment, Behind Express Hotel, Alkapuri, Vadodara, Gujarat 390007 India; 5 Ipas/Nepal, Ward No. 04, Do Cha Marg, Baluwatar, Kathmandu, Nepal; 6 Ipas/North Carolina, Technical Excellence Unit, PO Box 9990, Chapel Hill, NC 27515 USA; 7 CARE International in Uganda, P. O. Box 7280, 5th Floor, Union House. Plot 78, Luthuli Avenue - Bugolobi, Kampala, Uganda; 8 CARE USA, Global Advocacy Team, 115 Broadway Ave, 5th Floor, New York, NY 10006 USA

**Keywords:** Sexual and reproductive health, Social accountability, Adolescent, Abortion, Social exclusion

## Abstract

Social accountability is often put forward as a strategy to promote health rights, but we lack a programmatic evidence base on if, when, and how social accountability strategies can be used to promote access to quality Sexual and Reproductive Health (SRH) care for stigmatized populations and/or stigmatized issues. In this Commentary, we discuss the potential advantages and disadvantages of social accountability strategies in promoting the availability of a full range of SRH services for excluded and historically oppressed populations. We accomplish this by describing four programs that sought to promote access to quality SRH care for stigmatized populations and/or stigmatized services. Program implementers faced similar challenges, including stigma and harmful gender norms among providers and communities, and lack of clear guidance, authority, and knowledge of Sexual and Reproductive Health and Rights (SRHR) entitlements at local level. To overcome these challenges, the programs employed several strategies, including linking their strategies to legal accountability, budgetary expenditures, or other institutionalized processes; taking steps to ensure inclusion, including through consultation with excluded or stigmatized groups throughout the program design and implementation process; specific outreach and support to integrating marginalized groups into program activities; and the creation of separate spaces to ensure confidentiality and safety. The program experiences described here suggest some general principles for ensuring that social accountability efforts are inclusive both in terms of populations and issues addressed. Further empirical research can test and further flesh out these principles, and deepen our understanding of context.

## Background

Activists, Non-Governmental Organizations (NGOs), and donors point to social accountability as one key approach to improve the realization of Sexual and Reproductive Health and Rights (SRHR) related rights and goals. Broadly defined, social accountability refers to “ongoing and collective effort[s] to hold public officials to account for the provision of public goods which are existing state obligations” [1]. This paper is a reflection on social accountability practice in the domain of SRHR. We discuss the use of social accountability to promote access to quality Sexual and Reproductive Health (SRH) care for stigmatized populations and/or stigmatized services. We are concerned with SRH services where all who seek them may be stigmatized to some extent, such as abortion; populations who by nature of their identity may face discrimination in seeking any SRH service, such as lower caste populations or persons with disabilities; and, populations who are stigmatized due to their violating prevailing social norms in seeking a particular SRH service, such as adolescents seeking contraception. We raise key points from the theory and practice of social accountability to explore the ways in which common social accountability approaches may exclude stigmatized populations or issues. We go on to share relevant experiences from four programs, and to present factors for success and challenges these programs faced.

### Equity and inclusion in social accountability processes

Social accountability programs typically entail community actors assessing government performance against an agreed set of standards; a deliberative consensus building or priority setting element, wherein community members use these data to discuss and identify priorities; two-way dialogue between communities and the health system about these priorities; and follow up to ensure that these priorities are addressed. For example, a community group may compile a scorecard delineating key elements of the government maternal health strategy, assess gaps at their local health facilities, and, through a deliberative process, decide which gaps they wish to discuss with the government; or, jointly agree on budget priorities in a participatory budgeting or budget monitoring process [2–4]. Deliberative processes often yield widely shared priorities, potentially leaving out the specific needs of the few. In addition, prevailing power dynamics may serve to diminish or exclude certain voices. As described in the deep body of literature on community participation and the emerging evidence from studies on accountability for SRHR, deliberative processes may be dominated by members of the community who have the most power, marginalizing or tokenizing women, people with disabilities, young people, racial and ethnic minorities, lower caste populations, and other historically oppressed groups [5–7]. Moreover, everyone – but especially marginalized people - may face significant risk and repercussions in speaking out, such as health care providers refusing to treat them, violence in the home, or community censure [7–9]. Many program models for accountability fail to account for these hierarchies, such as by having facilitators who are skilled at supporting engagement from community members who feel unsafe or unable to speak [4, 10]. As a result, the priorities identified may not reflect the needs or priorities of those who are the most harmed by the status quo.

Few studies on social accountability for SRHR assess the inclusiveness of the social accountability process or the extent to which the outcomes favor equity, though some programs have gender or age specific groupings in order to encourage participation among marginalized people [11–13]. One study that set out to examine explicitly the extent to which pregnant adolescents participated in a community scorecard project focused on maternal health, found that the while pregnant and recently pregnant adolescents reported stigmatizing and rude treatment by health providers, they were unlikely to participate in or benefit from the community scorecard project [14]. The meetings were inconveniently timed, the adolescents felt uncomfortable discussing their own pregnancy, and the priorities arising from community meetings did not include their particular challenges [14]. On top of these concerns related to marginalized groups’ desire and ability to participate, there are also broader ethical concerns about expecting marginalized individuals to articulate their concerns in contexts where collective action among particular groups is unsafe and responsiveness by the state is unlikely [7].

### Stigma and politicization of sexual and reproductive health and rights

In addition to challenges in ensuring inclusion and equity in social accountability efforts generally, efforts regarding SRHR are shaped by the politicization, social mores, and stigma attached to SRHR in particular [13–15]. In the context of rising populism; a global, coordinated anti SRH movement; and the COVID-19 pandemic; stigmatization and hostility to SRHR is increasing in many contexts, contributing to a less enabling environment and extra challenges for civic engagement [16–18]. The health system is a social institution, reflecting these political and social dynamics characterizing the society at large [10, 19]. Values, norms, and judgements related to issues such as single motherhood, sexuality, and fecundity may influence provider and policy-maker attitudes regarding key SRHR issues, as well as the quality of care provided [15, 20]. Moreover, national law, policy, and health entitlements often reflect these broader social norms, such that the rights of particular populations – such as trans individuals – are not protected; access to SRH services and information, such as comprehensive sexual education or contraception for adolescents, is limited by law; or particular SRH services, such as abortion, are criminalized.

These social, political, and law and policy factors may shape which SRH issues are addressed in social accountability efforts. Given that most social accountability efforts aim to promote the realization of rights enshrined in national laws and policy, advocacy for SRHR that are enshrined in international human rights law but not national systems may be limited in many social accountability projects. The social norms and values of program implementers, participants, and likely respondents, such as health providers, may also influence which elements of SRHR are ultimately chosen as the focus of social accountability efforts. For example, a 2004 review of community participation in 18 World Bank-supported health reform projects in Asia found evidence suggesting that community participation translated into gains in maternal health and family planning services, but less – if any – impact on more stigmatized areas, such as Sexually Transmitted Infections (STIs), abortion services, and health services for violence survivors [21]. Similarly, a systematic review of accountability efforts for SRHR concluded that controversial issues tend to be given less priority, including contraception for adolescents, single women, and elderly people, and the SRH needs of sex workers [15].

We describe how four programs aimed to promote inclusion and equity, and navigated the stigma and politicization relating to SRHR in particular. The programs discussed were selected based on their stated intent to apply social accountability strategies to SRHR. The program successes and challenges presented were chosen following discussions among the authors; the content of each section is derived from the authors’ own program management experiences, as well as from internal and external evaluations. While we justify our statements with examples, this paper is in the style of a practice reflection or commentary, rather than a research study. Thus, the findings and principles we present are intended as hypotheses for further consideration by program implementers and exploration by researchers with expertise and engagement in social accountability and/or SRHR; we argue for fuller programmatic and research engagement with the topic. We also expect that this paper will contribute to broader discussions within the social accountability field about promoting inclusion.

## Program experiences

Table 1 summarizes the programs discussed.

**Table 1:** Social accountability programs addressing SRHR

Country and Implementing NGO	Key SRHR Focus	Population	Social Accountability Program Description
India, SAHAJ	SRHR information Access to adolescent friendly SRH services	Adolescents and young people – urban poor, tribal, rural, Dalit, living with disability, and sexually diverse peer leaders in selected districts of Gujarat State	Despite commitments made in the National Adolescents’ Health Programme and the Adolescent Reproductive and Sexual Health strategy of the Government of India, health services are generally not adolescent friendly. SAHAJ’s programme aims to support leadership development, inclusion and solidarity among adolescents and young people, in part so that they can effectively demand accountability form duty bearers. The community monitoring program engages adolescents, with special efforts to involve Dalit, LGBTQ+, and adolescents with disabilities. The adolescents use health service delivery monitoring tools, and hold dialogues and public hearings with service providers and district and state officers to discuss the findings of the community monitoring efforts. They also support participating adolescents and youth to develop relationships with other stakeholders to form coalitions for engagement in national planning fora.
Nepal, Ipas and local partners	Access to abortion and contraceptive services SRHR information for community and local government members	Women of reproductive age, young women, women living with disabilities, and adolescent girls in 2 rural districts Community members, local civil society organizations, and men	Pursuant to a policy of decentralization, the constitutionally protected right to health is now the primary responsibility of the local government. To ensure that access to SRHR, including safe abortion services, is fulfilled, Ipas Nepal is working with local civil society actors on a social accountability effort that supports and mobilizes women from the community to use community scorecards (a type of community monitoring), participatory planning and budgeting with local health authorities, and social audit (public hearings). These local civil society actors make particular efforts to engage adolescents and women/girls with disabilities.
Northern Uganda (humanitarian setting), CARE International in Uganda and Center for Reproductive Rights (CRR)	Access to comprehensive sexual and reproductive health and rights services and information	Refugee and host community women and girls of reproductive age (15–49 years) residing in one northern Uganda district	Human rights standards and principles apply in humanitarian situations, including the right to the highest attainable standard of SRH. Humanitarian actors and community women and girls in northern Uganda affirmed that existing accountability mechanisms (e.g. feedback boxes, hotlines) at refugee settlement and district level were inaccessible and/or unresponsive to issues relating to SRHR. Thus, CARE and CRR initiated a social accountability program with a three-pronged structure that includes a community council for SRHR, a third-party ombudsperson, and community monitors. The monitors collect SRHR complaints, which are then reviewed by the Council, and if needed, ombudsperson. The Council and ombudsperson engage duty-bearers (i.e. district government, refugee and local councils, and humanitarian health service providers) to ensure access to an effective remedy if rights to SRH are not respected.
Zambia, Copperbelt Province, Ipas and local partners	Youth access to contraceptive services	Youth members of youth-led and youth-serving organizations; SRHR clubs in tertiary education (male and female) in 2 districts	There is a high rate of unintended pregnancies among adolescents and youth in rural and urban areas of Zambia. Yet, adolescent SRH is not prioritized in National Health Strategic Plans and Budgets; and there is limited provision of adolescent-friendly SRH services. Moreover, adolescents and young people themselves have little knowledge of their SRHR entitlements and limited voice in public and governmental fora. Using a user-centered design process, Ipas facilitated the development of a community score card. The score card delineates governmental obligations related to adolescent SRHR. Members of youth organizations then monitor and document service delivery using these score cards, and dialogue with representatives of the local government to discuss results.

## Common challenges

In the programs we discuss, common challenges emerged that are similar to those encountered in other social accountability programs, including financing and budgetary constraints; risk of social and physical harm perpetrated by household members, community members, or health system actors who are threatened by the issues being raised and/or by the individuals mobilizing to raise them; and, inability to meaningfully address issues that are perceived to be beyond the authority of the program participants. Rather than focus on these more common challenges, we explore challenges that have particular relevance to ensuring comprehensive SRHR below.

### Stigma and harmful gender norms among providers and communities

All of the programs aimed to address stigma among providers, but, deeply embedded norms and beliefs about gender, reproduction, and sexuality posed a challenge. For example, youth engaged with the SAHAJ program in Gujarat, India, found it relatively easy to engage with the health system on issues related to nutrition, and they successfully ensured regular hemoglobin testing for adolescent girls to identify iron deficiency, as well as consistent access to weekly iron folic acid supplementation and take-home rations. However, they found it more difficult to communicate successfully to decision-makers and providers regarding adolescent sexual health, in part because providers seemingly held stigmatizing attitudes about adolescent sexuality and lacked knowledge of the public health evidence base in this area. For example, recent SAHAJ research found that a Community Health Worker (CHW) threatened to report a woman seeking information about abortion to her in-laws, and some CHWs expressed a desire to “control” young women who putatively exhibited sexuality in the “way they walk” [22].

Concerns about poor access to comprehensive abortion and post-abortion care services and information were raised during the program scoping of the CARE International in Uganda and Center for Reproductive Rights (CRR) program addressing refugee and host population women and girls in Northern Uganda. However, these issues have not come up in the Community Council or reported to the Ombudsperson. The program team speculates that this may be due to stigma about abortion among providers and communities.

Thus, irrespective of the anti-discrimination legislation in force or what entitlements are enshrined in law and policy, values and norms relating to SRHR may shape what issues are addressed as part of social accountability efforts, as well as if and how providers and governments respond. This is an unsurprising conclusion; shifting social norms that reflect longstanding social hierarchies entails a long process of contestation and debate [23]; while a social accountability program may advance that change, it is certainly insufficient to effect it.

### Lack of clear guidance, authority, and knowledge of SRH entitlements at local level

Government responsiveness is challenged in contexts where local level providers and officials lack clarity, knowledge, or resources to fulfill their responsibilities. Lack of capacity undermines many social accountability efforts, but it is possible that this challenge is greater for stigmatized issues and populations.

Nepal has recently decentralized its health system, and local governments have the authority and responsibility to manage health service budgets and service provision, including safe abortion services. In this new context, local governments were more responsive to community members and local NGOs assessing abortion service delivery as part of a social accountability program run by Ipas Nepal and local partners in two rural districts. Despite this, local Ministry of Health staff tasked with managing service delivery at the local level sometimes lacked the information and capacity required to fulfill their responsibilities. The Ministry of Health had instituted accountability mechanisms – such as a citizens’ charter and health facility operation and management committees, but awareness of these mechanisms within communities was poor. An Ipas program evaluation revealed that knowledge of these mechanisms increased significantly in the areas where the social accountability program is being implemented as compared to a control, but still, 38% of community members did not know about these mechanisms in program areas; the percentage was even higher in comparison districts at 62%.

SAHAJ has realized that some front-line service providers in program districts in India lack important knowledge or skills regarding adolescents’ sexuality related concerns, including menstruation; SAHAJ team members have thus begun to train them on adolescent SRHR issues. Building on this, peer leaders have used the training as a basis to identify what they feel is lacking in service delivery, including support for boys seeking advice on nocturnal emissions and STIs.

Limited knowledge of the application of human rights standards and principles was a key feature among stakeholders in the Northern Uganda CARE/CRR program. At the onset of the program, few refugee and host women and girls understood their access to SRH services as entitlements, or experiences of discrimination as violations that require accountability and access to effective remedies. Lack of clarity and knowledge about entitlements were particularly stark for stigmatized issues such as adolescent access to contraception and access to comprehensive post-abortion care regardless of the legal status of abortion.

None of the programs we represent explicitly sought to ascertain whether or not local level authority and knowledge regarding SRHR entitlements differed from those regarding other entitlements. Nonetheless, our experience suggests that this may be the case, particularly for stigmatized issues. Explicit attention to addressing this frontline knowledge gap may be especially important for social accountability efforts addressing SRHR.

## Common strategies for success

The implementers of these programs raised many factors for success that are commonly identified in the peer reviewed research on social accountability, such as garnering support for a social accountability program from the communities involved, health system actors, and local politicians. In addition to these findings that are ubiquitous in the literature, we highlight two broad groupings of strategies below that our programs employ that are especially relevant to comprehensive SRHR for all.

### Link to legal accountability, budgetary expenditures, or other institutionalized processes to promote tangible changes to communities

Social accountability efforts have grappled with key questions about how to make sure that duty bearers respond [24–26]. Grounding programs in public administration or legal processes can provide institutional traction; this may be particularly helpful in the case of stigmatized issues or populations that may not be easily accorded priority.

In Northern Uganda, integrating legal accountability strategies at all stages of program consultation, design, implementation, and monitoring provided the program duty-bearers and rights holders with a clear rationale for the program and clarified their respective SRHR responsibilities and entitlements. This was accomplished through an accessible and ongoing translation of the complementary bodies of law that apply in humanitarian settings [27] and those that Uganda has ratified at international and regional levels, and legislated at national levels. Complaints filed with the program’s Community Council for SRHR were analyzed through a clear framework of what constitutes an SRHR violation, and, if it was established a right had been violated, the associated remedy. The complaints protocol included a procedure for prioritizing the problems affecting the most marginalized; the Council has addressed issues such as unavailable health commodities for STI treatment; denials of antiretroviral treatments for new refugees arriving in the settlement; inaccessibility and unacceptability of medical equipment for women with disabilities; and disrespect and abuse experienced by refugee women seeking antenatal care.

Similarly, in India, SAHAJ has used existing government policies and norms as the framework for community-led research in order to spur governmental responsiveness. Program participants documented service availability and quality, using the resultant data to point out gaps in the operationalization of governmental programs. For example, recent SAHAJ research described the prevalence of poor knowledge about contraception amongst working class urban young women, despite concerted government efforts to increase access [22]. In addition to access for some groups of women, quality of services is also an issue in India. SAHAJ and partners documented the deaths of poor tribal women in sterilization camps in central India, revealing that the government was failing to ensure standards for sterilization care laid down by the Supreme Court [28].

Local NGOs working with Ipas in Nepal promoted the implementation of comprehensive SRH programs by engaging directly in the budget process. They approached local government bodies to advocate for the inclusion of safe abortion services in the budget, and to institutionalize this inclusion. As a result, the local health system had the resources to meet their obligation to ensure access to free comprehensive abortion care. The governments used the funds allocated in the budget to train service providers; to raise awareness about SRH entitlements among girls by providing sessions on reproductive health and rights - including abortion - in schools; and to directly fund abortion services.

In Zambia, the Ipas and local partners’ social accountability program seeking to address barriers to contraception access among youth in the Copperbelt Province utilized Neighborhood Health Committees. Neighborhood Health Committees are pre-existing quasi-governmental structures that include health providers, community health workers, and community members, including youth and individuals participating in local Safe Motherhood Action Groups. Youth participants in the social accountability program appreciated that this structure offered access to Ministry of Health representatives and service providers. Moreover, youth advocates advocated for improved access to contraception through frameworks that the Ministry had committed to, namely evidence-based public health and Ministry commitments regarding contraception. The program complemented this approach with strategic engagement with other Ministry of Health actors, namely designated Adolescent Focal Points in health facilities and central level officials.

Reliance on existing state obligations is a defining feature of social accountability. In the case of individuals and SRHR issues that are subject to stigma or discrimination, reference to these obligations might be particularly important, perhaps because they can be used to communicate government priorities to previously unaware providers, or because they can deflect blame or lessen space for discretion, e.g. “I am just following the rules.” Similarly, social accountability programs successfully pushing for the integration of SRHR into existing state processes can confer legitimacy and sustainability to the services concerned. In brief, despite the fact that government policies and programs and public sector employee behaviors are shaped by wider social norms, in many contexts, formal rules and processes may allow for activities that prioritize public health evidence rather than restrictive social norms. This strategy would not work in cases where laws, policies, and institutional processes themselves exclude services or populations, such as laws that criminalize LGBT populations or restrict abortion.

### Intentional inclusivity by creating distinct roles and groups for marginalized populations

In contrast to approaches that engage communities as geographically defined (e.g. residents of a particular village), the programs described here focused on particularly marginalized groups, such as adolescents, or made special efforts to engage these groups. There has been limited research on this approach to social accountability, but the existing research suggests that social accountability programs could be more rights-based by ensuring that each component of the program, such as information gathering, mobilization, and engagement, takes explicit steps to support participation of excluded groups [4].

The CARE Uganda/CRR program in Northern Uganda includes a specific adolescent representative on the program established Community Council; this person has raised several adolescent specific concerns, including menstrual hygiene kit inaccessibility for refugee girls, and discrimination and stigma experienced by pregnant adolescents when seeking antenatal care at the health center. The adolescent representative also established an adolescent specific solidarity group to discuss concerns that the broader Community Council should address. This separate space for dialogue and sharing accurate SRH information helped participants to feel more comfortable discussing intimate issues, and helped to protect adolescent participants’ privacy and confidentiality, making it less likely that they would experience reprisals for raising stigmatized SRHR questions.

Youth were involved from the program design stage in Zambia, influencing the social accountability program priorities and outcomes. They identified community score card indicators for success, as well as the scoring procedures, and included youth-friendly services in the score card. According to an internal midline assessment, the program has contributed to increased knowledge of youth and parents regarding available contraceptive services, the establishment of youth-friendly corners in health facilities, commitments from the Ministry of Health to provide transportation for adolescents to the clinic, improved availability of contraceptive options at facilities, and longer service hours in facilities. Importantly, the groups of adolescents from the four facilities who participated in the program educated both service providers and policymakers about adolescents’ needs and challenges.

In Nepal, the community score card program included standalone, confidential meetings with women who had recently obtained abortions. Identified by local civil society organizations, these women discussed the quality of care received, including the behavior of health providers, overall treatment by the health service, and availability of medication abortion and sanitation facilities at the point of care.

The SAHAJ program included outreach to sexual minority youth and adolescents – and then, once these groups felt comfortable, integrating them into their more general youth social accountability work. This has expanded other youths’ awareness of exclusion and expanded solidarity, culminating in the 2019 Pride March, when there was a large turnout of new allies.

In sum, the programs took three main approaches to inclusion: (1) consultation with excluded or stigmatized groups throughout the program design and implementation process, (2) specific outreach and support to integrating marginalized groups into program activities, and (3) the creation of separate spaces to ensure confidentiality and safety, safeguarding emotional, social, and physical security. These efforts fostered environments where existing power hierarchies can be transgressed, and create the conditions for the inclusion of historically excluded people and SRH issues into social accountability efforts.

## Conclusion

Realization of the right to sexual and reproductive health requires providing comprehensive, high quality information and services to all people, especially those who are excluded. While a robust evidence base on if, when, and how social accountability strategies can be used to promote this goal is lacking, the program experiences described here suggest some general principles for ensuring that social accountability efforts are inclusive both in terms of populations and issues addressed. First, in the context of stigma and discrimination, it is perhaps even more important than for programs addressing less stigmatized issues and people, that efforts rely on legal and other formalized commitments, obligations, and rationales. Invoking these formal commitments may create space to surface unacknowledged persons and issues, making them issues of acceptable public concern. Second, inclusion should be built into the program, and permeate all stages of implementation. Depending on the context, this may require creating specific processes for excluded groups. Further empirical research can test and further flesh out these principles and hypotheses, and deepen our understanding of context.

## Data Availability

Not applicable.

## Abbreviations

CHW: Community Health Worker
CRR: Center for Reproductive Rights
NGO: Non-governmental Organization
SRH: Sexual and Reproductive Health
SRHR: Sexual and Reproductive Health and Rights
STI: Sexually Transmitted Infection
